# Effect of endometrial thickness on obstetric and neonatal outcomes in assisted reproduction: a systematic review and meta-analysis

**DOI:** 10.1186/s12958-023-01105-6

**Published:** 2023-06-13

**Authors:** Zheng Fang, Jialyu Huang, Jiaqin Mao, Lamei Yu, Xiaohong Wang

**Affiliations:** 1 Department of Gynecology and Obstetrics, Reproductive Medical Center, Tangdu Hospital, Air Force Medical University, Xi’an, China; 2 Reproductive Medical Center, Jiangxi Maternal and Child Health Hospital, Nanchang Medical College, Nanchang, China; 3 Department of Obstetrics, Jiangxi Maternal and Child Health Hospital, Nanchang Medical College, Nanchang, China

**Keywords:** Endometrial thickness, Obstetric outcome, Neonatal outcome, Meta-analysis

## Abstract

**Purpose:**

This systematic review and meta-analysis aimed to explore the relationship of endometrial thickness (EMT) with obstetric and neonatal outcomes in assisted reproductive cycles.

**Methods:**

PubMed, EMBASE, Cochrane Library and Web of Science were searched for eligible studies through April 2023. Obstetric outcomes include placenta previa, placental abruption, hypertensive disorders of pregnancy (HDP), gestational diabetes mellitus (GDM) and cesarean section (CS). Neonatal outcomes include birthweight, low birth weight (LBW), gestational age (GA), preterm birth (PTB), small for gestational age (SGA) and large for gestational age (LGA). The effect size was estimated as odds ratio (OR) or mean difference (MD) with 95% confidence interval (CI) using a random-effects model. Inter-study heterogeneity was assessed by the chi-square homogeneity test. One-study removal method was used to determine the sensitivity of the meta-analysis.

**Results:**

Nineteen studies involving 76,404 cycles were included. The pooled results revealed significant differences between the thin endometrium group and the normal group in placental abruption (OR = 2.45, 95% CI: 1.11–5.38, *P* = 0.03; I^2^ = 0%), HDP (OR = 1.72, 95% CI: 1.44–2.05, *P* < 0.0001; I^2^ = 0%), CS (OR = 1.33, 95% CI: 1.06–1.67, *P* = 0.01; I^2^ = 77%), GA (MD = -1.27 day, 95% CI: -2.41– -1.02, *P* = 0.03; I^2^ = 73%), PTB (OR = 1.56, 95% CI: 1.34–1.81, *P* < 0.0001; I^2^ = 33%), birthweight (MD = -78.88 g, 95% CI: -115.79– -41.98, *P* < 0.0001; I^2^ = 48%), LBW (OR = 1.84, 95% CI: 1.52–2.22, *P* < 0.00001; I^2^ = 3%) and SGA (OR = 1.41, 95% CI: 1.17–1.70, P = 0.0003; I^2^ = 15%). No statistical differences were found in placenta previa, GDM, and LGA.

**Conclusion:**

Thin endometrium was associated with lower birthweight or GA and higher risks of placental abruption, HDP, CS, PTB, LBW and SGA. Therefore, these pregnancies need special attention and close follow-up by obstetricians. Due to the limited number of included studies, further studies are needed to confirm the results.

**Supplementary Information:**

The online version contains supplementary material available at 10.1186/s12958-023-01105-6.

## Introduction

Since the birth of the first test-tube baby, assisted reproductive technology (ART) has brought hope to many infertile families. However, with the deepening of research, emerging studies have found that there are potential safety issues in ART pregnancy [1–4], such as low birth weight (LBW), preterm birth (PTB) and hypertensive disorders of pregnancy (HDP). At present, the mechanisms remain unclear and complex.

A thin endometrium is of great concern in ART cycles. Endometrial thickness (EMT) can be measured through a convenient way by transvaginal ultrasound and less than 7 or 8 mm is generally considered to be thin [5]. Although patients with thin endometrium can achieve and maintain a pregnancy spontaneously, these patients are reported to have significantly lower biochemical pregnancy, implantation and live birth rates during the process of ART [6, 7]. Furthermore, recent studies have revealed an association of thin endometrium with adverse obstetric and neonatal outcomes. However, no consensus has been reached and the relevance is still controversial [8–29]. Many factors may lead to this controversy, such as the type of embryo transfer, different cut-off values of EMT, and the number of cases reported in the study. Therefore, we conducted this systematic review and meta-analysis to determine associations between EMT and ART cycle outcomes to shed further light on this question.

## Materials and methods

### Protocol and registration

We conducted and reported our review based on the Preferred Reporting Items for Systematic Reviews and Meta-Analysis Statement (PRISMA2020) [30]. The study protocol is accessible at https://www.crd.york.ac.uk/PROSPERO/ (registration number CRD42021273323) while we excluded ectopic pregnancy in this study.

### Data sources, search strategy and selection criteria

The electronic databases PubMed, Cochrane Library, Embase and Web of Science were searched until April 2023 for articles which evaluated effect of EMT on obstetric and neonatal outcomes in assisted reproduction. The selection criteria were described according to Patients, Intervention, Comparison and Outcomes (PICO) statements. Briefly, we included infertile women who had singleton livebirths after undergoing in vitro fertilization/ intracytoplasmic sperm injection (IVF/ICSI) or intra-uterine insemination (IUI) cycles. Patients were divided into the thin (intervention) and normal (comparison) groups based on the EMT cut-off values referring to the original studies. EMT was defined as the maximal distance between one interface of endometrium– myometrium to the other and measured according to corresponding cycles (Table 1). The outcomes included obstetric outcomes (placenta previa, placental abruption, HDP, gestational diabetes mellitus [GDM] and cesarean section [CS]) as well as neonatal outcomes (birthweight, LBW, gestational age [GA], PTB, small for gestational age [SGA] and large for gestational age [LGA]) defined according to International Classification of Diseases (ICD)-10 codes. Studies were excluded if: (1) studies were published as a letter, abstract or case report; (2) studies were not published in English; (3) samples were duplicated; and (4) samples were less than 20.

**Table 1:** Main characteristics of included studies in the systematic review and meta-analysis

Study	Country	Study design	No. of cycles	Mean age	Cut-off value	Treatment	Type of embryo transfer	Cycle protocol	Day of EMT measurement	NOS score^a^
Hu 2021 [12]	China	Retrospective cohort	5220	30.2	8.0 mm	IVF/ICSI	Frozen	artificial cycle, natural cycle, ovulation induction cycle	hCG trigger or progesterone initiation day	9
Guo 2020 [10]	China	Retrospective cohort	3157	31.5	7.5 mm	IVF/ICSI	Fresh	GnRH agonist, GnRH antagonist, mild stimulation, natural cycle	hCG trigger day	8
He 2019 [11]	China	Retrospective cohort	1139	30.5	8.0 mm	IVF/ICSI	Fresh and frozen	NA	hCG trigger or progesterone initiation day	7
Borges 2019 [8]	Brazil	Retrospective cohort	402	34.1	NA	ICSI	Fresh	GnRH antagonist	NA	8
Oron 2018 [17]	Israel	Retrospective cohort	864	32.5	7.5 mm	IVF/ICSI	Fresh	GnRH agonist, GnRH antagonist, natural cycle	hCG trigger day	7
Kaser 2015 [15]	US	Case–control	199	NA	9.7 mm	IVF/ICSI	Fresh and frozen	Fresh: NA Frozen: artificial cycle	NA	9
Chung 2006 [9]	US	Case–control	435	31.9	10 mm	IVF/ICSI	NA	NA	The last recorded thickness prior to oocyte retrieval	7
Liu 2021 [20]	China	Retrospective cohort	9266	30.9	8.0 mm	IVF/ICSI	Fresh	GnRH agonist, GnRH antagonist, other	hCG trigger day	8
Huang 2020 [13]	China	Retrospective cohort	1016	30.2	7.6 mm	IUI	NA	Letrozole + HMG	hCG trigger day	9
Ribeiro 2018 [18]	Belgium	Retrospective cohort	939	NA	7.0 mm	IVF/ICSI	Fresh	GnRH antagonist	hCG trigger day	8
Jing 2019 [14]	China	Retrospective cohort	5251	30.9	9.0 mm	IVF/ICSI	Frozen	Artificial cycle, natural cycle	the day before embryo thawing	9
Zhang 2019 [19]	China	Retrospective cohort	6181	32.0	8.0 mm	IVF/ICSI	Frozen	Artificial cycle, natural cycle	hCG trigger or progesterone initiation day	9
Moffat 2017 [16]	Switzerland	Retrospective cohort	764	34.4	NA	IVF/ICSI	Fresh	GnRH agonist, GnRH antagonist	hCG trigger day	9
Rombauts 2014 [21]	Australia	Retrospective cohort	4537	34.4	9.0 mm	IVF/ICSI	Fresh and frozen	Fresh: GnRH agonist, GnRH antagonist Frozen: artificial cycle, natural cycle	hCG trigger or progesterone initiation day	8
Huang 2021 [23]	China	Retrospective cohort	1755	30.0	8.0 mm	IVF/ICSI	Frozen	GnRH agonist, GnRH antagonist	hCG trigger or progesterone initiation day	8
Liu 2021a [25]	China	Retrospective cohort	9273	31.2	8.0 mm	IVF/ICSI	Fresh	GnRH agonist, GnRH antagonist, other	hCG trigger day	7
Zhang 2022 [28]	China	Retrospective cohort	13,458	30.5	8.0 mm	IVF/ICSI	Frozen	Natural cycle, artificial cycle	hCG trigger or progesterone initiation day	7
Zheng 2022 [29]	China	Retrospective cohort	4313	NA	8.0 mm	IVF/ICSI	Frozen	Artificial cycle, natural cycle	progesterone initiation day	7
He 2022 [22]	China	Retrospective cohort	8235	29.4	7.5 mm	IVF/ICSI	Frozen	Natural cycle, artificial cycle	hCG trigger or progesterone initiation day	9

^a^Study with scores greater than 7 was regarded as high quality. *GnRH* gonadotrophin releasing hormone, *HMG* human menopausal gonadotrophin, *ICSI* intracytoplasmic sperm injection, *IUI* intrauterine insemination, *IVF* in vitro fertilization, *NA* not available, *NOS* Newcastle–Ottawa Scale

The following keywords and their synonyms were used for literature search: [(‘endometrial thickness’) and (‘IVF’ or ‘ICSI’ or ‘infertility treatment’ or ‘IUI’ or ‘assisted reproductive technology’) and (‘pregnancy complications’ or ‘infant, newborn, diseases’ or ‘neonatal outcome’)] (see Supplementary File 1 for full strategy). Titles and abstracts of all identified studies were screened and the full paper of the preselected articles was scrutinized by two researchers (Z.F. and J.Q.M.). Any disagreement was settled by a third author (J.L.H.) to make the final decision.

### Data collection and quality assessment

Two independent authors (Z.F. and J.Q.M.) extracted data from eligible studies by using standardized extraction forms. The following variables were collected: first author’s surname, publication year, country, study design, number of cycles, mean age, cut-off value of EMT, treatment, type of embryo transfer, cycle protocol, and obstetric and neonatal outcomes in the corresponding EMT groups. If 2 × 2 tables could be constructed, the study was selected for meta-analysis. If not, the study was selected for systematic review. In the 2 × 2 tables, the number of cycles with obstetric complications or reported neonatal outcomes for different EMT cut-off values was recorded. Authors were contacted by email if information was missing. Any disagreement between the two researchers was resolved through discussion, or in case of persistent disagreement, by consultation with a third author (J.L.H.).

Study quality was assessed by two researchers (Z.F. and J.Q.M.) using the Newcastle–Ottawa Scale (NOS) [31] based on selection, comparability and exposure (case–control study) or outcomes (cohort study).The score of a study below 6 signifies low quality, 6 and 7 represents moderate quality, while 8 and 9 means good quality. Any inconsistencies between the two authors were adjudicated by an additional author (X.H.W.) referring to the original article.

### Statistical analysis

The pooled data for investigated outcomes were calculated using the random-effects model, considering that the underlying effects varied across the studies included [32, 33]. The incidences of placenta previa, placental abruption, HDP, GDM, PTB, LBW, SGA, LGA and CS were assigned as dichotomous data, and the odds ratios (ORs) with 95% confidence intervals (CIs) were calculated. The GA and birthweight were assigned as continuous variables, and mean differences (MDs) were calculated between the groups to determine the effect size [34].

Chi-squared homogeneity test and Higgins index (I^2^) were applied to evaluate the heterogeneity of articles. Heterogeneity was regarded as: none (I^2^ < 25%), low (25% ≤ I^2^ < 50%), moderate (25% ≤ I^2^ < 75%), or high (I^2^ > 75%). To assess the impact of a single study on the outcome, one-study removal method was used to determine the sensitivity of the meta-analysis [35]. Since not enough studies (fewer than ten) were included in the pooled analysis, the assessment of publication bias was not conducted according to Cochrane Handbook recommendations [14]. Subgroup analyses for HDP, GDM, SGA, PTB and LBW were conducted based on type of embryo.

RevMan software (Review Manager, version 5.4) was used for all statistical analyses conducted in this study. All tests were two tailed and a *P*-value of less than 0.05 was deemed statistically significant.

## Result

### Literature search and selection

**Fig. 1:**
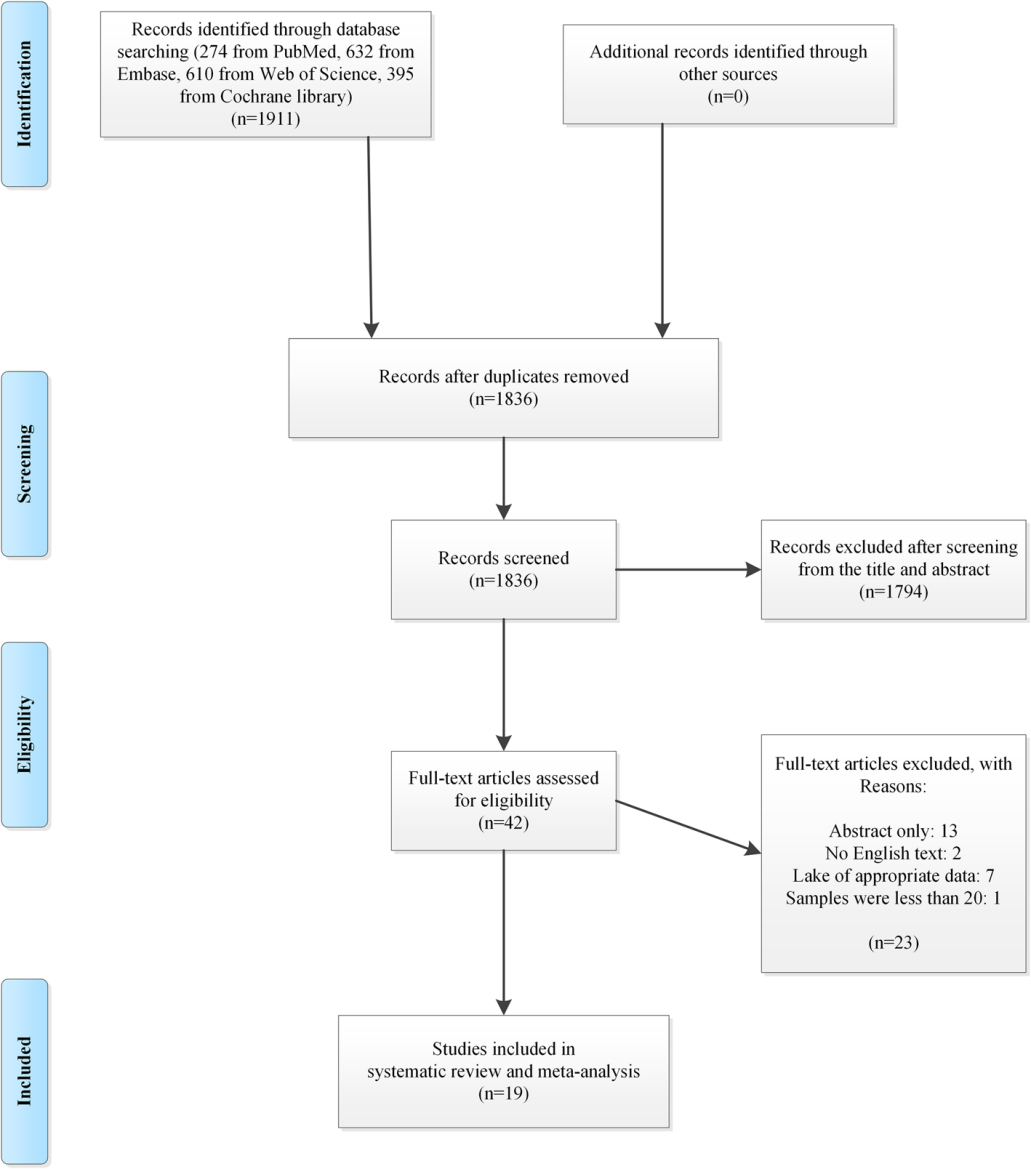
The flow diagram of study selection

The search strategy identified a total of 1911 articles. After removing duplicates, 1836 abstracts were reviewed, and 42 full-text articles were further assessed. Thirteen studies were published as conference abstract, two studies were not English, and seven studies only reported live birth rate without obstetric or neonatal outcomes. In addition, the study by Martel et al*.* [36] was excluded as only 7 patients were enrolled in the thin endometrium group. Finally, 19 articles were appropriate to be included in this systematic review and meta-analysis (Fig. 1) [8–23, 25, 28, 29].

The characteristics of all 19 included studies are presented in Table 1. Among them, 17 were retrospective cohort studies and 2 were case–control studies. The study sample size ranged from 199 to 13,458 cycles, for a total of 76,404 cycles. Studies were published between 2006 and 2022, and participants were mainly from China. EMT was divided into dichotomous variables in 15 studies [10–14, 17–21], which were thus included in meta-analysis. Most studies defined thin endometrium as EMT below 8 mm [11, 12, 19, 20, 23, 25, 28, 29], while different cut-off values of 7.0, 7.5, 7.6 and 9.0 mm were used in other studies [10, 13, 14, 17, 21, 22]. Some of the data in Liu et al. [20] were partially duplicated with those in Guo et al. [10], and we retained the study containing a larger sample size during the analysis. The remaining four studies analyzed EMT as a continuous variable and were included in the systematic review as data could not be extracted. Overall, the included studies were at low risk of bias with a NOS score of 7 (six studies), 8 (six studies) or 9 (seven studies), with details shown in Table S1.

### Systematic review

Borges and colleagues analyzed the effect of EMT on birthweight of 402 newborns and showed that EMT was positively correlated with birthweight (β = 28.351, P = 0.044) and was significantly lower in the SGA group compared to the normal group [8]. Similarly, by analyzing 764 fresh cycles, Moffat et al*.* found that the EMT could predict neonatal birthweight [16].The study conducted by Chung and colleagues analyzed the effect of EMT on PTB, LBW and intrauterine fetal demise by comparing 159 cases and 276 controls [9]. The study showed a two-fold overall increased risk in the EMT ≤ 10 mm group compared to the EMT > 12 mm group (OR = 2.04, 95% CI: 1.09–3.83). With each millimeter increase in EMT, the risk of adverse perinatal outcome could decrease by 12%.

In addition, Kaser et al*.* conducted a case–control study analyzing 50 placenta accreta cases and 149 controls [15]. The study demonstrated that in cryopreserved embryo transfer cycles, the accreta patients had a significantly lower EMT than non-accreta patients.

### Meta-analysis of obstetric outcomes

#### Placenta previa

**Fig. 2:**
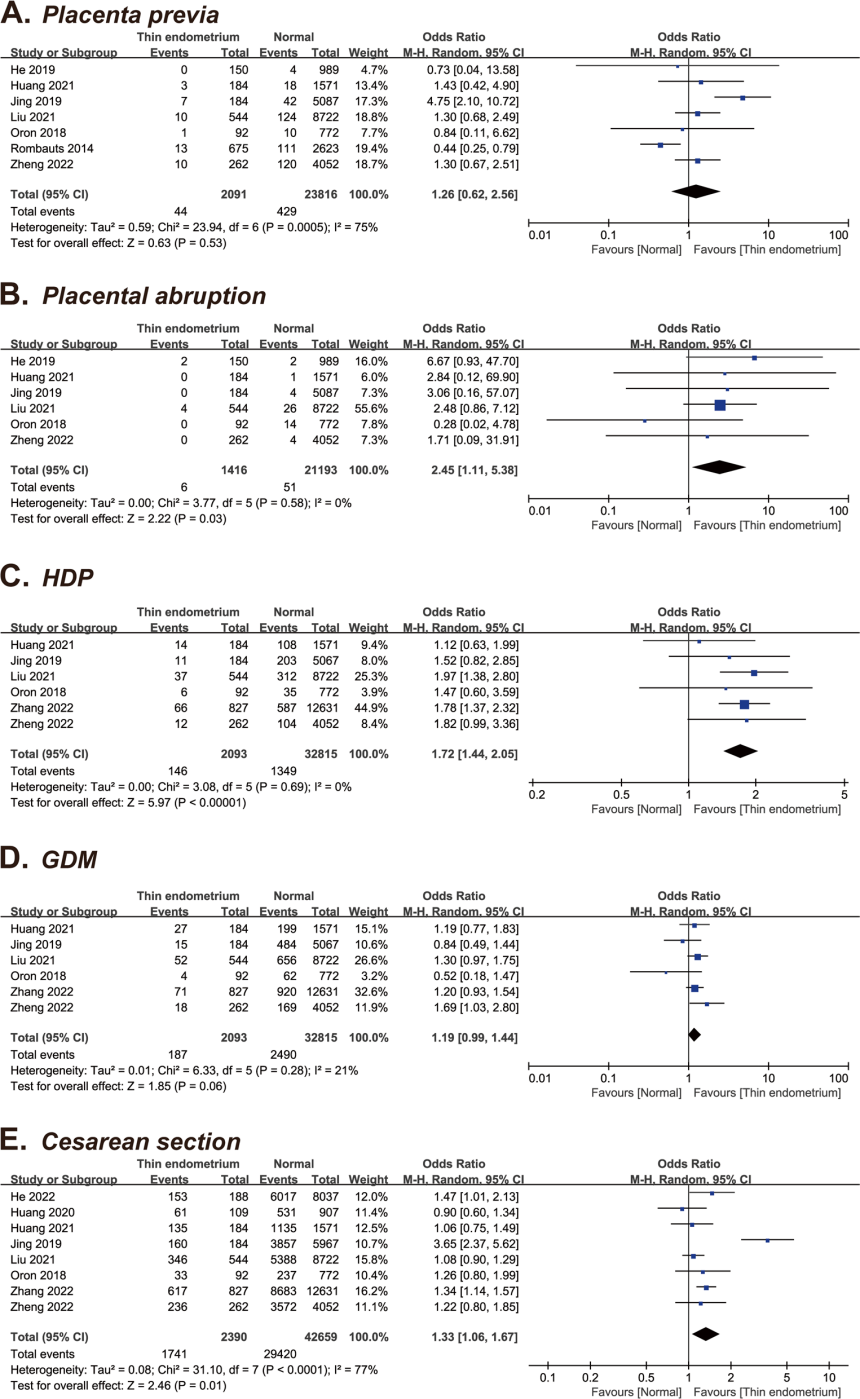
Forest plots of obstetric outcomes for thin versus normal endometrium. **A** Placenta previa; **B** Placental abruption; **C** Hypertensive disorders of pregnancy; **D** Gestational diabetes mellitus; **E** Cesarean section

Seven studies [11, 14, 17, 20, 21, 23, 29], including 25,907 patients, reported on placenta previa rate. Pooled analysis revealed that thin endometrium was not associated with the risk of placenta previa (OR = 1.26, 95% CI: 0.62–2.56, *P* = 0.53; I^2^ = 75%) (Fig. 2A).

#### Placental abruption

Six studies [11, 14, 17, 20, 23, 29], including 22,609 patients, reported on placental abruption. The overall OR for placental abruption was 2.45 (95% CI: 1.11–5.38, *P* = 0.03; I^2^ = 0%), suggesting no significant difference between the thin and normal endometrium groups (Fig. 2B).

#### Hypertensive disorders of pregnancy

Six studies [14, 17, 20, 23, 28, 29], including 34,908 patients, were pooled in this meta-analysis. Overall, the risk of HDP was significantly higher in the thin endometrium group than in the normal EMT group (OR = 1.72, 95% CI: 1.44–2.05, *P* < 0.0001; I^2^ = 0%) (Fig. 2C).

#### Gestational diabetes mellitus

Six studies [14, 17, 20, 23, 28, 29], including 34,908 patients, were combined in this analysis. Overall, no difference was noted in the GDM risk between the thin endometrium group and the normal endometrium groups (OR = 1.19, 95% CI: 0.99–1.44, *P* = 0.06; I^2^ = 21%) (Fig. 2D).

#### Cesarean section

Eight studies [13, 14, 17, 20, 22, 23, 28, 29], including 45,049 patients, were pooled in this analysis. Compared with the normal EMT group, the thin endometrium group showed a higher incidence of cesarean section (OR = 1.33, 95% CI: 1.06–1.67, *P* = 0.01) and the heterogeneity was high (I^2^ = 77%) (Fig. 2E).

### Meta-analysis of neonatal outcomes

#### Gestational age

**Fig. 3:**
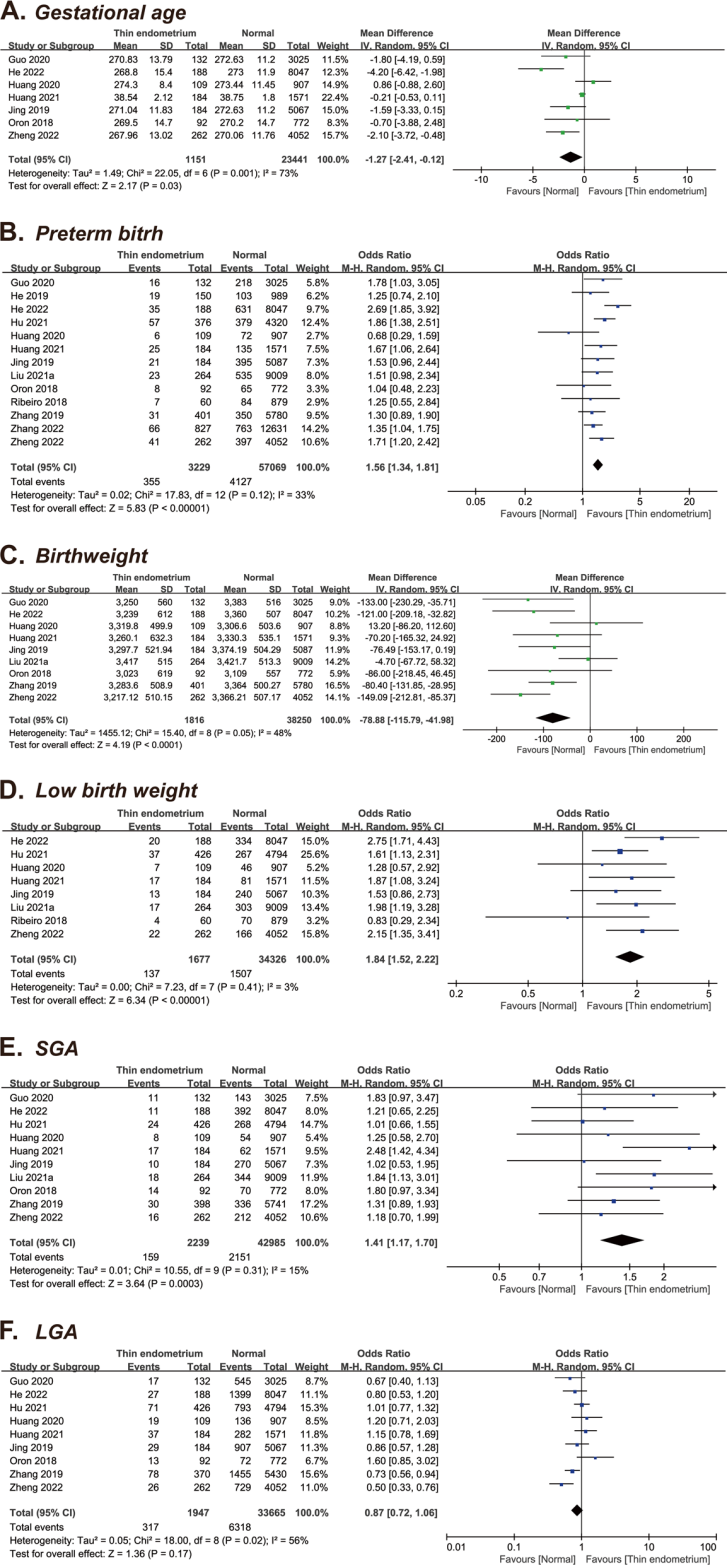
Forest plots of neonatal outcomes for thin versus normal endometrium. **A** Gestational age; **B** Preterm birth; **C** Birthweight; **D** Low birth weight; **E** Small for gestational age; **F** Large for gestational age

Seven studies [10, 13, 14, 17, 22, 23, 29], including 24,592 patients, were part of this analysis. Overall, decrease was noted in the GA between the thin endometrium and the normal EMT groups (MD = -1.27 days, 95% CI: -2.41–0.12, *P* = 0.03), with a high heterogeneity (I^2^ = 73%) (Fig. 3A).

#### Preterm birth

Thirteen studies [10–14, 17–19, 22, 23, 25, 28, 29], including 60,298 patients, provided information on PTB, which allowed quantitative pooled analysis. A significantly higher risk of PTB was found in the thin endometrium group relative to the normal endometrium group (OR = 1.56, 95% CI: 1.34–1.81, *P* < 0.0001; I^2^ = 33%) (Fig. 3B).

#### Birthweight

Nine studies [10, 13, 14, 17, 19, 22, 23, 25, 29], which included 40,066 patients, provided data on the birthweight. Overall, the thin endometrium group showed a significantly lower birthweight than the normal endometrium group (MD = -78.88 g, 95% CI: -115.79– -41.98, *P* < 0.0001; I^2^ = 48%) (Fig. 3C).

#### Low birthweight

Eight studies [12–14, 18, 22, 23, 25, 29], including 36,003 patients, were analyzed. Overall, the risk of LBW was significantly higher in the thin endometrium group than in the normal EMT group (OR = 1.84, 95% CI: 1.52–2.22, *P* < 0.00001; I^2^ = 3%) (Fig. 3D).

#### Small for gestational age

Ten studies [10, 12–14, 17, 19, 22, 23, 25, 28, 29], including 45,224 patients, were pooled in this analysis. When comparing the thin endometrium group to the normal EMT group, the OR for SGA was 1.41 (95% CI: 1.17–1.70, *P* = 0.0003) with low heterogeneity (I^2^ = 0%) (Fig. 3E).

#### Large for gestational age

Nine studies [10, 12–14, 17, 19, 22, 23, 29], including 35,612 patients, evaluated the LGA outcome. No significant difference was noted in the LGA incidence between the thin endometrium group and the normal EMT group (OR = 0.87, 95% CI: 0.72–1.06, *P* = 0.17; I^2^ = 56%) (Fig. 3F).

### Sensitivity analysis

On excluding the study by Jing et al*.* or Oron et al*.*, pooled analysis revealed that thin endometrium was associated with significantly higher risk of GDM (excluded Jing et al*.*: OR = 1.25, 95% CI: 1.04–1.49, *P* = 0.02; excluded Oron et al*.*: OR = 1.23, 95% CI: 1.05–1.44, *P* = 0.01). Contrarily, removal of any other individual studies did not modify the pooled estimates significantly in other obstetric and neonatal outcomes.

## Discussion

### Principal findings

The results of this meta-analysis showed that the HDP, CS, PTB, LBW and SGA risks were significantly higher in the thin endometrium group while neonatal birthweight and GA were significantly lower in the thin endometrium group. There were no significant differences in other maternal and perinatal outcomes between the two groups.

### Interpretation of the findings

Previous meta-analyses have been conducted to investigate the association between EMT and pregnancy outcomes following IVF/ICSI treatment. It was generally concluded that thin endometrium could lead to lower rates of implantation, clinical pregnancy, ongoing pregnancy and live birth [5, 7]. Previous meta-analysis showed thin endometrium leads to a higher incidence of HDP and a lower birth weight, while a thick endometrium had no influence on pregnancy, maternal, or perinatal outcomes [24], compared to this study, we included more studies and outcomes. In the present study, we demonstrate the first-time systematic evidence that decreased EMT is also linked with increased obstetric and neonatal complications, indicating that long-term healthcare should be provided for these women even in cases of successful pregnancy.

In the subgroup analysis, outcomes were conducted based on type of embryo. Previous studies have shown that FET was associated with higher risk of HDP, LGA while lower risk of placenta previa, placental abruption, LBW, PTB and SGA [26]. We found that the subgroup analysis did not change the original results except for LBW of fresh cycles, this may be a bias due to the small number of studies (Figure S1, S2, S3, S4 and S5).

In sensitivity analyses, most findings remained coincident when one study was removed at a time, implying the reliability of our meta-analyses. Nonetheless, removal of the study by Jing et al*.* or Oron et al*.* resulted in a significant change of the pooled estimate of GDM [14, 17]. Both studies detected no difference in GDM rate between groups. However, the cut-off of EMT was 9.0 mm in Jing’s study, which may cause bias compared to other studies. Therefore, this inconsistent result may be attributed to the limited sample size or cut-off values that differed from other studies, and further large cohorts should be performed for confirmation.

### Biological plausibility

HDP is a common pregnancy complication and a major contributor to PTB, LBW and SGA. However, the etiology of HDP has not yet been fully elucidated [37]. HDP is usually associated with uteroplacental hypoperfusion and ischemia, a common pathophysiologic mechanism also shared by placental abruption and intrauterine growth restriction. During the formation of the placenta, extravillous trophoblast cells invade the inner third of the uterine myometrium, replace the spiral artery endothelium, cause the collapse of vascular smooth muscles and thus remodel the blood vessels in this area. After vascular remodeling, a low-resistance blood flow connection is established between the spiral artery and the uterine radial artery, consequently increasing the circulating blood volume in the intervillous space and the placenta to provide nutrients for the growth and development of the fetus [38]. However, this process may be disordered in patients with decreased EMT. Indeed, an important feature of thin endometrium is the increased resistance of the uterine radial artery, which affects the normal placental blood supply [39]. In addition, studies have found that factors related to angiogenesis, such as leukemia inhibitory factor (LIF), vascular endothelial growth factor (VEGF) and β3 integrin are insufficient or even completely absent [40]. In this regard, endothelial cells could lack stimulation of pro-angiogenic factors during the remodeling process, resulting in reduced blood supply and leading to HDP or adverse neonatal outcomes [41].

Another mechanism may lie in the difference of oxygen tension between thin and normal endometrium. Under normal circumstances, the spiral arteries would constrict after ovulation, and the blood flow on the endometrial surface is reduced [42], creating a local low oxygen concentration that is conducive to successful embryo implantation. However, in patients with thin endometrium, the placenta and the fetus may be closer to the basal endometrium with greater blood flow and an oxygen-rich environment [43]. As a result, more free radicals are generated, thus possibly leading to impaired fetal growth [44].

In addition to the above, decreased EMT may be associated with certain ART process, thus leading to higher risks. For example, a previous meta-analysis showed that artificial cycles could lead to higher HDP and PTB rate [27]. In most of included studies, the endometrial preparation protocols were significantly different between normal and thin endometrium groups. Therefore, this may lead to a difference in obstetric and neonatal outcomes.

### Strengths and limitations

To our knowledge, this systematic review and meta-analysis is the first study to comprehensively evaluate the relationship between thin endometrium and adverse obstetric and neonatal outcomes. Of the 14 included studies, 11 were published in recent five years. Hence, the effects of publication year and associated technical change on the pooled analysis were greatly reduced. Sensitivity analyses were performed by one-study removal method to assess the robustness of pooled data. Moreover, this meta-analysis was performed strictly according to the PRISMA 2020 statement and therefore, the quality of the methodology and reporting is high.

This study has some limitations. First and most importantly, all included studies are retrospective cohort studies or case–control studies with inherent bias. Analysis was based on crude data instead of adjusted data, and lack of prospective studies may lead to overestimation or underestimation of results. Secondly, due to different cut-off values of included studies, we cannot find a unified EMT to analyze. Thirdly, this study was based on small number of studies in each outcome, which limited our further conduction of subgroup analyses according to type of embryo transfer and number of cycles. Other unavoidable biases, such as the inclusion of only articles published in English and the exclusion of conference abstracts, may also have affected the results.

## Conclusion

Our meta-analysis showed that patients with thin endometrium may face higher risks of certain adverse obstetric and neonatal outcomes compared to those with normal endometrium. This finding could provide useful information for both clinicians and infertile patients. Effective treatment should be provided to these women to increase EMT during ART cycles, while continuous monitoring and follow-up are needed throughout pregnancy. Given the present limitations, more prospective cohort studies with larger sample size are warranted to confirm the conclusions.

## Supplementary Information

**Additional file 1: Table S1.** Quality assessment of included studies by the Newcastle–Ottawa scale.

**Additional file 2: Figure S1.** Subgroup analyses for HDP based on type of embryo.

**Additional file 3: Figure S2.** Subgroup analyses for GDM based on type of embryo.

**Additional file 4: Figure S3.** Subgroup analyses for SGA based on type of embryo.

**Additional file 5: Figure S4.** Subgroup analyses for PTB based on type of embryo.

**Additional file 6: Figure S5.** Subgroup analyses for LBW based on type of embryo.

**Additional file 7: **Search strategy

## Data Availability

The original data from the survey is available.

## Abbreviations

ART: Assisted reproductive technology
CI: Confidence interval
CS: Cesarean section
EMT: Endometrial thickness
GA: Gestational age
GDM: Gestational diabetes mellitus
HDP: Hypertensive disorders of pregnancy
IUI: Intra-uterine insemination
IVF/ICSI: In vitro fertilization/ intracytoplasmic sperm injection
LBW: Low birth weight
LIF: Leukemia inhibitory factor
LGA: Large for gestational age
MD: Mean difference
NOS: Newcastle–Ottawa Scale
OR: Odds ratio
PICO: Patients, Intervention, Comparison and Outcomes
PRISMA: Preferred Reporting Items for Systematic Reviews and Meta-Analysis Statement
PTB: Preterm birth
SGA: Small for gestational age
VEGF: Vascular endothelial growth factor
