# Supplementary material for: Effect of endometrial thickness on obstetric and neonatal outcomes in assisted reproduction: a systematic review and meta-analysis
Source: Reprod Biol Endocrinol. 2023 Jun 13;21:55. doi: 10.1186/s12958-023-01105-6 (PMC10262454; doi:10.1186/s12958-023-01105-6)
Supplement: Supplementary file 1 — Additional file 1: Table S1. Quality assessment of included studies by the Newcastle–Ottawa scale. [file 12958_2023_1105_MOESM1_ESM.docx]

**Table S1.** Quality assessment of included studies by the Newcastle-Ottawa scale.

|  | Representativeness of the exposed cohort | Selection of the non-exposed cohort | Ascertainment of exposure | | Absence of outcome at start | Comparability on design or analysis | Assessment of outcome | Duration of follow-up | Adequacy of follow-up | Total score |
| --- | --- | --- | --- | --- | --- | --- | --- | --- | --- | --- |
| Hu *et al.*, 2021 | ★ | ★ | ★ | ★ | | ★★ | ★ | ★ | ★ | 9 |
| Guo *et al.*, 2020 | ★ | ★ | ★ | ★ | | ★★ | ★ | ★ | ○ | 8^a^ |
| He *et al.*, 2019 | ★ | ★ | ★ | ★ | | ★○ | ★ | ★ | ○ | 7^b^ |
| Borges *et al.*, 2019 | ★ | ★ | ★ | ★ | | ★★ | ★ | ★ | ○ | 8^a^ |
| Oron *et al.*, 2018 | ★ | ★ | ★ | ★ | | ○○ | ★ | ★ | ★ | 7^c^ |
| Liu *et al.*, 2021 | ★ | ★ | ★ | ★ | | ★○ | ★ | ★ | ★ | 8^d^ |
| Huang *et al.*, 2020 | ★ | ★ | ★ | ★ | | ★★ | ★ | ★ | ★ | 9 |
| Ribeiro *et al.*, 2018 | ★ | ★ | ★ | ★ | | ★○ | ★ | ★ | ★ | 8^e^ |
| Jing *et al.*, 2019 | ★ | ★ | ★ | ★ | | ★★ | ★ | ★ | ★ | 9 |
| Zhang *et al.*, 2019 | ★ | ★ | ★ | ★ | | ★★ | ★ | ★ | ★ | 9 |
| Moffat *et al.*, 2017 | ★ | ★ | ★ | ★ | | ★★ | ★ | ★ | ★ | 9 |
| Rombauts *et al.*, 2014 | ★ | ★ | ★ | ★ | | ★○ | ★ | ★ | ★ | 8^f^ |
| Huang *et al.*, 2021 | ★ | ★ | ★ | ★ | | ★★ | ★ | ★ | ○ | 8^a^ |
| Liu *et al.*, 2021a | ★ | ★ | ★ | ★ | | ★○ | ★ | ★ | ○ | 7^g^ |
| Zhang *et al.*, 2022 | ★ | ★ | ★ | ★ | | ★○ | ★ | ★ | ○ | 7^g^ |
| Zheng *et al.*, 2022 | ★ | ★ | ★ | ★ | | ○○ | ★ | ★ | ★ | 7^c^ |
| He *et al.*, 2022 | ★ | ★ | ★ | ★ | | ★★ | ★ | ★ | ★ | 9 |
|  | Case definition | Representativeness of the cases | Selection of controls | Definition of controls | | Comparability of cases and controls | Ascertainment of exposure | Same method of ascertainment | Non-response rate | Total score |
| Kaser *et al.*, 2015 | ★ | ★ | ★ | ★ | | ★○ | ★ | ★ | ★ | 9 |
| Chung *et al.*, 2006 | ★ | ★ | ★ | ★ | | ○○ | ★ | ★ | ★ | 7^b^ |

★, score = 1; ○, score = 0.

^a^ Lost one score because the study did not clearly describe the number of excluded cases and the status of loss to follow-up.

^b^ Lost two scores because 1) the study did not distinguish fresh and frozen-thawed embryo transfer cycle; 2) Adequacy of follow-up not described.

^c^ Lost two scores because the maternal age of two groups was significant and did not calculate the adjusted odd ratio.

^d^ Lost one score because the cause of infertility is significantly different.

^e^ Lost one score because the study did not describe the age of the groups.

^f^ Lost one score because the study did not distinguish fresh and frozen-thawed embryo transfer cycle.

^g^ Lost two scores because 1) the maternal age of two groups was significantly different; 2) the study did not clearly describe the number of excluded cases and the status of loss to follow-up.
