# Supplementary figures and images for: Effect of endometrial thickness on obstetric and neonatal outcomes in assisted reproduction: a systematic review and meta-analysis
Source: Reprod Biol Endocrinol. 2023 Jun 13;21:55. doi: 10.1186/s12958-023-01105-6 (PMC10262454; doi:10.1186/s12958-023-01105-6)

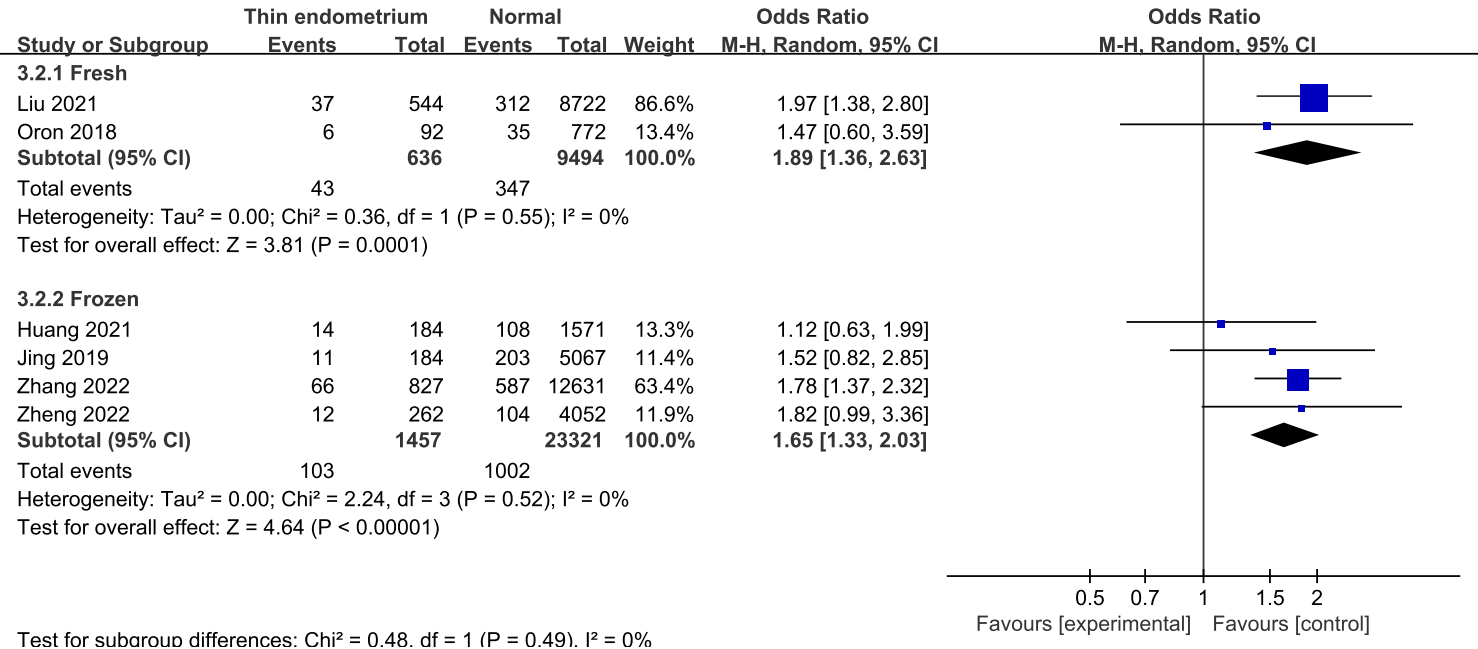

Supplement: Supplementary file 2 — Additional file 2: Figure S1. Subgroup analyses for HDP based on type of embryo. [file 12958_2023_1105_MOESM2_ESM.pdf]

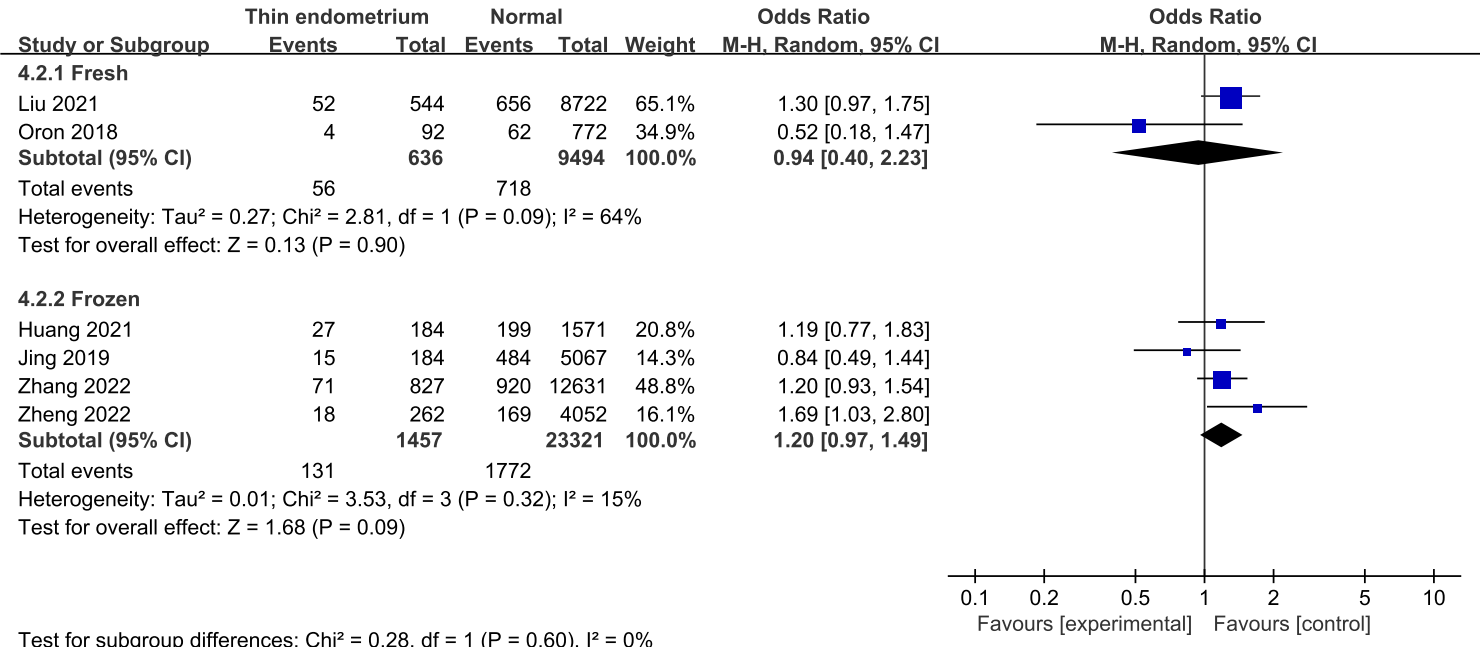

Supplement: Supplementary file 3 — Additional file 3: Figure S2. Subgroup analyses for GDM based on type of embryo. [file 12958_2023_1105_MOESM3_ESM.pdf]

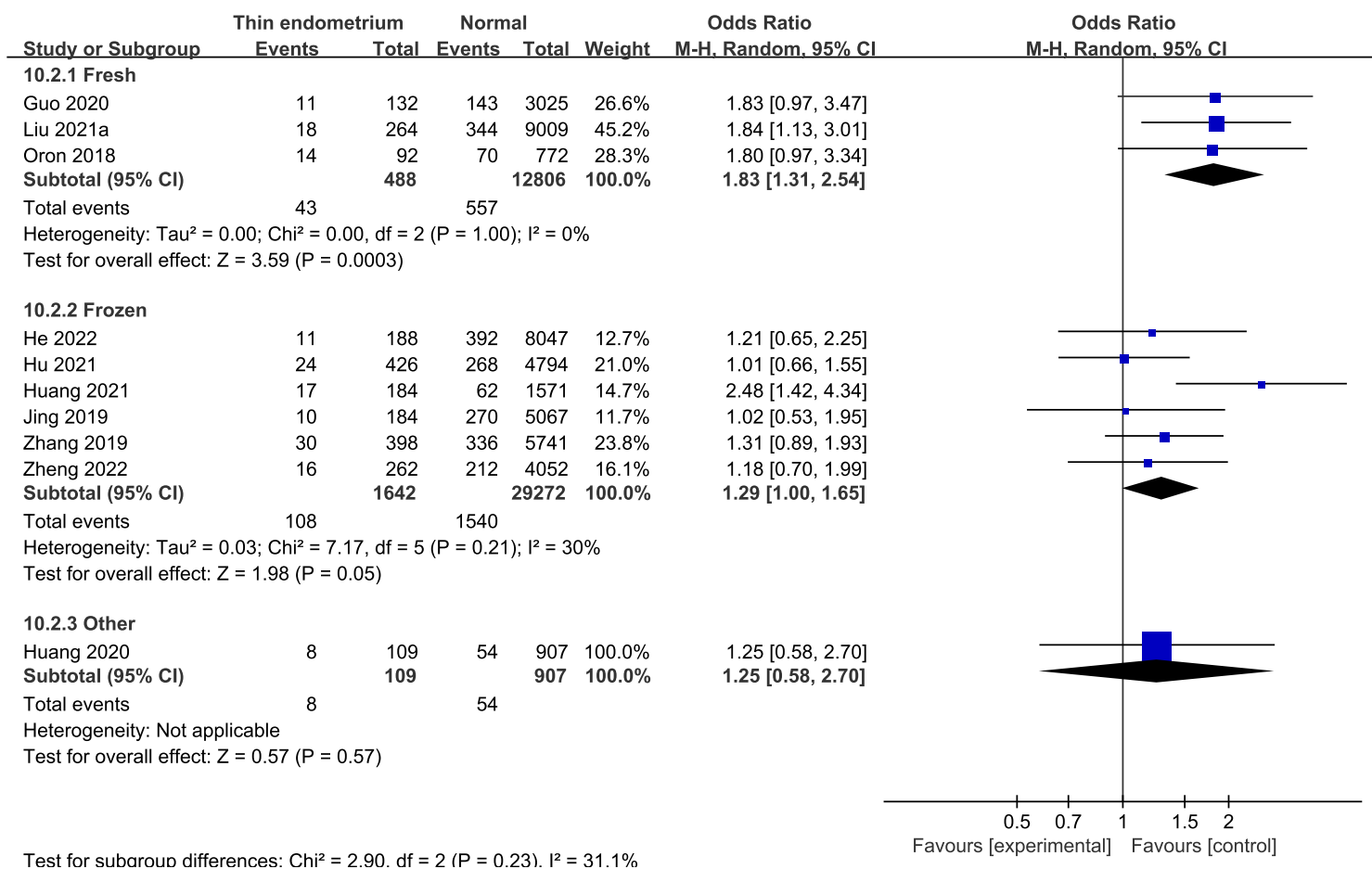

Supplement: Supplementary file 4 — Additional file 4: Figure S3. Subgroup analyses for SGA based on type of embryo. [file 12958_2023_1105_MOESM4_ESM.pdf]

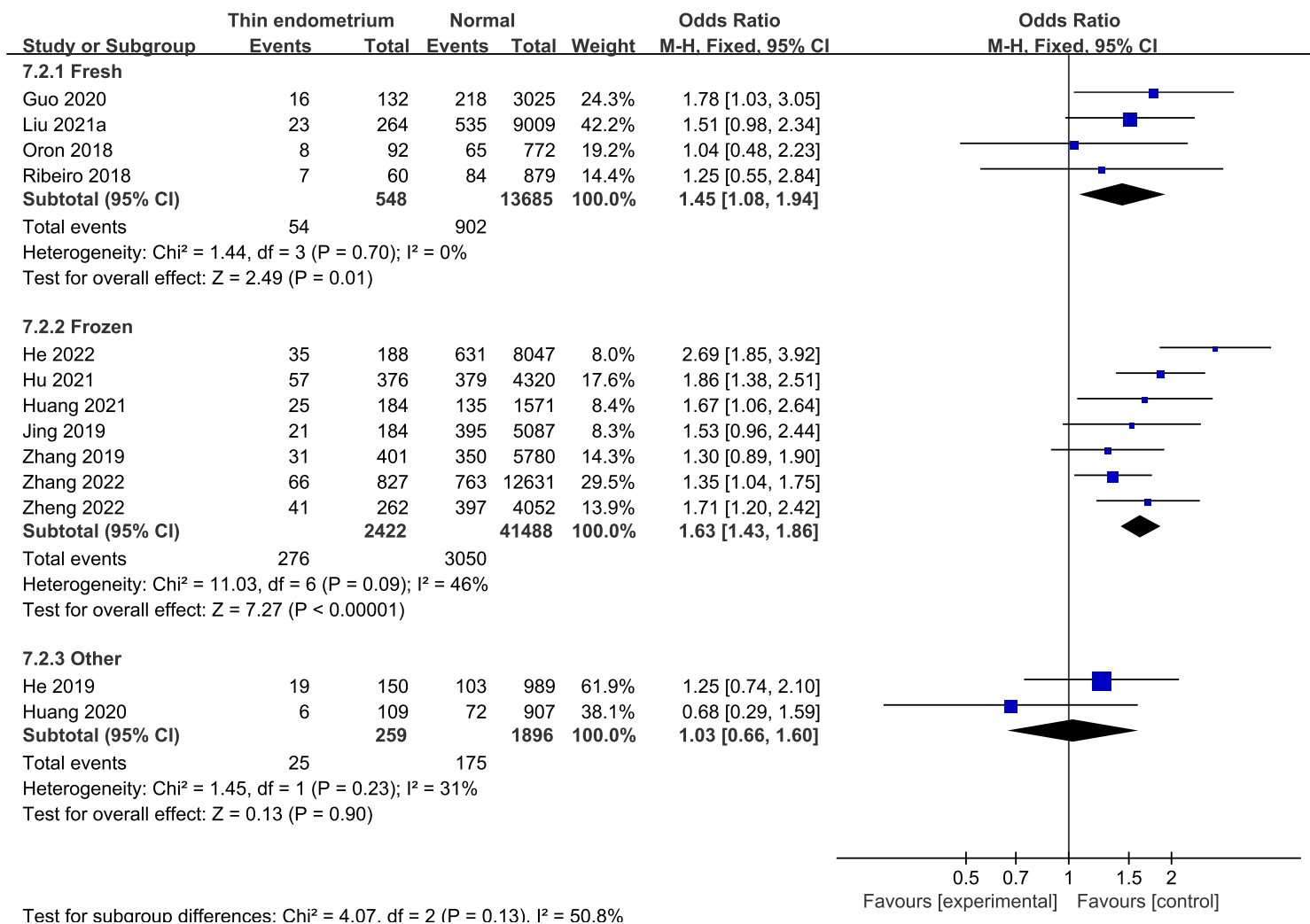

Supplement: Supplementary file 5 — Additional file 5: Figure S4. Subgroup analyses for PTB based on type of embryo. [file 12958_2023_1105_MOESM5_ESM.pdf]

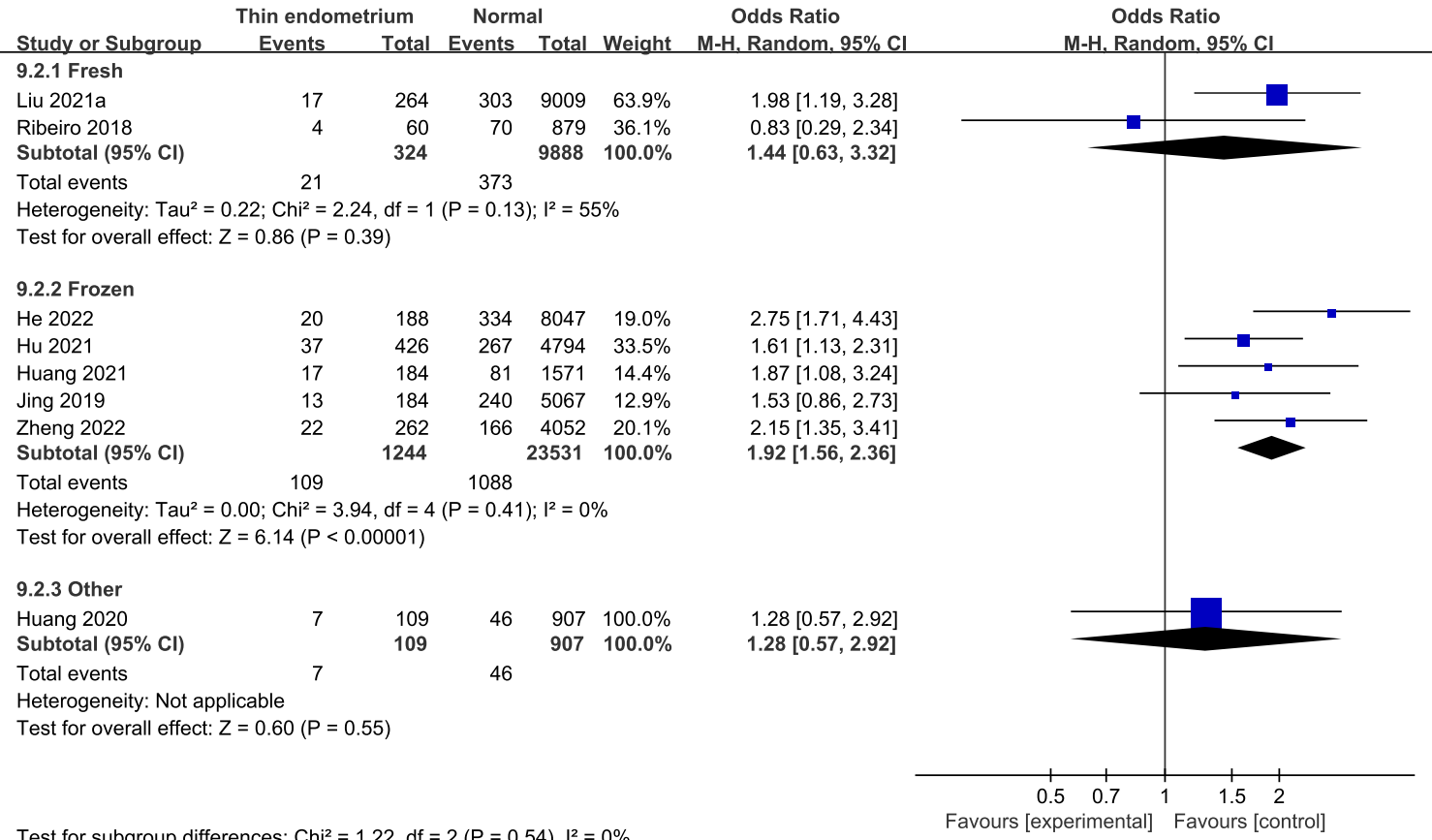

Test for subgroup differences: Chi² = 1.22. df = 2 (P = 0.54). I² = 0%

Supplement: Supplementary file 6 — Additional file 6: Figure S5. Subgroup analyses for LBW based on type of embryo. [file 12958_2023_1105_MOESM6_ESM.pdf]
