# Supplementary material for: Effect of endometrial thickness on obstetric and neonatal outcomes in assisted reproduction: a systematic review and meta-analysis
Source: Reprod Biol Endocrinol. 2023 Jun 13;21:55. doi: 10.1186/s12958-023-01105-6 (PMC10262454; doi:10.1186/s12958-023-01105-6)
Supplement: Supplementary file 7 — Additional file 7: Search strategy [file 12958_2023_1105_MOESM7_ESM.zip › PUBMED search strategy.docx]

总检索

(("endometrial thickness"[Title/Abstract] OR "endometrial pattern*"[Title/Abstract] OR "thin endometrium"[Title/Abstract] OR "endometrial ultrasound"[Title/Abstract] OR "endometrial ultrasonography"[Title/Abstract] OR "endometrial receptivity"[Title/Abstract] OR "endometrial characteristic*"[Title/Abstract] OR "thinner endometrium"[Title/Abstract] OR "suboptimal endometrial development"[Title/Abstract] OR "endometrial development"[Title/Abstract] OR "endometrial thicknesses"[Title/Abstract] OR "endometrial stripe"[Title/Abstract] OR "endometrial stripe thickness"[Title/Abstract] OR "endometrial lining"[Title/Abstract] OR "endometrial lining thickness"[Title/Abstract]) AND ((((((Sperm Injections, Intracytoplasmic[Mesh]) OR (((((((Injection, Intracytoplasmic Sperm[Title/Abstract]) OR (Injections, Intracytoplasmic Sperm[Title/Abstract])) OR (Intracytoplasmic Sperm Injection[Title/Abstract])) OR (Sperm Injection, Intracytoplasmic[Title/Abstract])) OR (Intracytoplasmic Sperm Injections[Title/Abstract])) OR (ICSI[Title/Abstract])) OR (Injections, Sperm, Intracytoplasmic[Title/Abstract]))) OR ((Sperm Injections, Intracytoplasmic[Mesh]) OR (((((((Injection, Intracytoplasmic Sperm[Title/Abstract]) OR (Injections, Intracytoplasmic Sperm[Title/Abstract])) OR (Intracytoplasmic Sperm Injection[Title/Abstract])) OR (Sperm Injection, Intracytoplasmic[Title/Abstract])) OR (Intracytoplasmic Sperm Injections[Title/Abstract])) OR (ICSI[Title/Abstract])) OR (Injections, Sperm, Intracytoplasmic[Title/Abstract])))) OR ((((((((((((((((((Assisted Reproductive Technique[Title/Abstract]) OR (Reproductive Technique, Assisted[Title/Abstract])) OR (Technique, Assisted Reproductive[Title/Abstract])) OR (Techniques, Assisted Reproductive[Title/Abstract])) OR (Assisted Reproductive Technics[Title/Abstract])) OR (Assisted Reproductive Technic[Title/Abstract])) OR (Reproductive Technic, Assisted[Title/Abstract])) OR (Reproductive Technics, Assisted[Title/Abstract])) OR (Technic, Assisted Reproductive[Title/Abstract])) OR (Technics, Assisted Reproductive[Title/Abstract])) OR (Assisted Reproductive Techniques[Title/Abstract])) OR (Reproductive Technology, Assisted[Title/Abstract])) OR (Assisted Reproductive Technologies[Title/Abstract])) OR (Assisted Reproductive Technology[Title/Abstract])) OR (Reproductive Technologies, Assisted[Title/Abstract])) OR (Technologies, Assisted Reproductive[Title/Abstract])) OR (Technology, Assisted Reproductive[Title/Abstract])) OR (Reproductive Techniques, Assisted[Mesh]))) OR ((Fertilization in Vitro[Mesh]) OR ((((((((((((In Vitro Fertilization[Title/Abstract]) OR (In Vitro Fertilizations[Title/Abstract])) OR (Test-Tube Fertilization Fertilization, Test-Tube[Title/Abstract])) OR (Fertilizations, Test-Tube[Title/Abstract])) OR (Test Tube Fertilization[Title/Abstract])) OR (Test-Tube Fertilizations[Title/Abstract])) OR (Fertilizations in Vitro[Title/Abstract])) OR (Test-Tube Babies[Title/Abstract])) OR (Babies, Test-Tube[Title/Abstract])) OR (Baby, Test-Tube[Title/Abstract])) OR (Test Tube Babies[Title/Abstract])) OR (Test-Tube Baby[Title/Abstract])))) OR ((((((Insemination, Artificial[MeSH Terms]) OR (Eutelegenesis[Title/Abstract])) OR (Eutelegeneses[Title/Abstract])) OR (Artificial Insemination[Title/Abstract])) OR (Artificial Inseminations[Title/Abstract])) OR (Inseminations, Artificial[Title/Abstract])))) AND (("pregnancy complications"[MeSH Terms] OR "complication pregnancy"[Title/Abstract] OR "pregnancy complication"[Title/Abstract] OR "complications pregnancy"[Title/Abstract] OR "adverse pregnancy outcome"[Title/Abstract] OR "obstetric outcome"[Title/Abstract] OR "obstetric complication"[Title/Abstract] OR "hypertensive disorders of pregnancy"[Title/Abstract] OR "pregnancy induced hypertension"[Title/Abstract] OR "pre-eclampsia"[Title/Abstract] OR "gestational diabetes mellitus"[Title/Abstract] OR "postpartum hemorrhage"[Title/Abstract] OR "cesarean section"[Title/Abstract] OR "placenta previa"[Title/Abstract] OR "placental abruption"[Title/Abstract] OR "placenta accreta"[Title/Abstract] OR "Cholestasis"[Title/Abstract]) OR ("infant, newborn, diseases"[MeSH Terms] OR "neonatal outcome"[Title/Abstract] OR "perinatal outcome"[Title/Abstract] OR "neonatal death"[Title/Abstract] OR "perinatal death"[Title/Abstract] OR "neonatal morbidity"[Title/Abstract] OR "perinatal morbidity"[Title/Abstract] OR "neonatal mortality"[Title/Abstract] OR "perinatal mortality"[Title/Abstract] OR "fetal growth"[Title/Abstract] OR "growth restriction"[Title/Abstract] OR "birth weight"[Title/Abstract] OR "Birthweight"[Title/Abstract] OR "gestational age"[Title/Abstract] OR "gestational week"[Title/Abstract] OR "preterm delivery"[Title/Abstract] OR "preterm birth"[Title/Abstract] OR "Macrosomia"[Title/Abstract] OR "small for gestational age"[Title/Abstract] OR "larger for gestational age"[Title/Abstract]))
