# Supplementary material for: Effect of endometrial thickness on obstetric and neonatal outcomes in assisted reproduction: a systematic review and meta-analysis
Source: Reprod Biol Endocrinol. 2023 Jun 13;21:55. doi: 10.1186/s12958-023-01105-6 (PMC10262454; doi:10.1186/s12958-023-01105-6)
Supplement: Supplementary file 7 — Additional file 7: Search strategy [file 12958_2023_1105_MOESM7_ESM.zip › Web of Science search strategy.docx]

1：TI=(endometrial thickness OR endometrial pattern* OR thin endometrium OR endometrial ultrasound OR endometrial ultrasonography OR endometrial receptivity OR endometrial characteristic* OR thinner endometrium OR suboptimal endometrial development OR endometrial development OR endometrial thicknesses OR endometrial stripe OR endometrial stripe thickness OR endometrial lining OR endometrial lining thickness OR embryo implantation) OR AB=(endometrial thickness OR endometrial pattern* OR thin endometrium OR endometrial ultrasound OR endometrial ultrasonography OR endometrial receptivity OR endometrial characteristic* OR thinner endometrium OR suboptimal endometrial development OR endometrial development OR endometrial thicknesses OR endometrial stripe OR endometrial stripe thickness OR endometrial lining OR endometrial lining thickness OR embryo implantation)

2：TI=(Pregnancy Complications OR Complication, Pregnancy OR Pregnancy Complication OR Complications, Pregnancy OR adverse pregnancy outcome OR Obstetric outcome OR Obstetric complication OR hypertensive disorders of pregnancy OR Pregnancy-induced hypertension OR pre-eclampsia OR gestational diabetes mellitus OR postpartum haemorrhage hemorrhage OR caesarean cesarean section OR placenta previa praevia OR placental abruption OR placenta accreta OR Cholestasis) OR AB=(Pregnancy Complications OR Complication, Pregnancy OR Pregnancy Complication OR Complications, Pregnancy OR adverse pregnancy outcome OR Obstetric outcome OR Obstetric complication OR hypertensive disorders of pregnancy OR Pregnancy-induced hypertension OR pre-eclampsia OR gestational diabetes mellitus OR postpartum haemorrhage hemorrhage OR caesarean cesarean section OR placenta previa praevia OR placental abruption OR placenta accreta OR Cholestasis)

3：TI=(Infant, Newborn, Diseases OR Neonatal outcome OR Perinatal outcome OR Neonatal death OR Perinatal death OR Neonatal morbidity OR Perinatal morbidity OR Neonatal mortality OR Perinatal mortality OR fetal growth OR growth restriction OR Birth weight OR Birthweight OR Gestational age OR Gestational week OR Preterm delivery OR Preterm birth OR Macrosomia OR small-for-gestational age OR larger-for-gestational age) OR AB=(Infant, Newborn, Diseases OR Neonatal outcome OR Perinatal outcome OR Neonatal death OR Perinatal death OR Neonatal morbidity OR Perinatal morbidity OR Neonatal mortality OR Perinatal mortality OR fetal growth OR growth restriction OR Birth weight OR Birthweight OR Gestational age OR Gestational week OR Preterm delivery OR Preterm birth OR Macrosomia OR small-for-gestational age OR larger-for-gestational age)

4：TI=(Sperm Injections, Intracytoplasmic OR Injection, Intracytoplasmic Sperm OR Injections, Intracytoplasmic Sperm OR Intracytoplasmic Sperm Injection OR Sperm Injection, Intracytoplasmic OR Intracytoplasmic Sperm Injections OR ICSI OR Injections, Sperm, Intracytoplasmic) OR AB=(Sperm Injections, Intracytoplasmic OR Injection, Intracytoplasmic Sperm OR Injections, Intracytoplasmic Sperm OR Intracytoplasmic Sperm Injection OR Sperm Injection, Intracytoplasmic OR Intracytoplasmic Sperm Injections OR ICSI OR Injections, Sperm, Intracytoplasmic)

5：TI=(Reproductive Techniques, Assisted OR Assisted Reproductive Technique OR Reproductive Technique, Assisted OR Technique, Assisted Reproductive OR Techniques, Assisted Reproductive OR Assisted Reproductive Technics OR Assisted Reproductive Technic OR Reproductive Technic, Assisted OR Reproductive Technics, Assisted OR Technic, Assisted Reproductive OR Technics, Assisted Reproductive OR Assisted Reproductive Techniques OR Reproductive Technology, Assisted OR Assisted Reproductive Technologies OR Assisted Reproductive Technology OR Reproductive Technologies, Assisted OR Technologies, Assisted Reproductive OR Technology, Assisted Reproductive) OR AB=(Reproductive Techniques, Assisted OR Assisted Reproductive Technique OR Reproductive Technique, Assisted OR Technique, Assisted Reproductive OR Techniques, Assisted Reproductive OR Assisted Reproductive Technics OR Assisted Reproductive Technic OR Reproductive Technic, Assisted OR Reproductive Technics, Assisted OR Technic, Assisted Reproductive OR Technics, Assisted Reproductive OR Assisted Reproductive Techniques OR Reproductive Technology, Assisted OR Assisted Reproductive Technologies OR Assisted Reproductive Technology OR Reproductive Technologies, Assisted OR Technologies, Assisted Reproductive OR Technology, Assisted Reproductive)

6：TI=(Fertilization in Vitro OR In Vitro Fertilization OR In Vitro Fertilizations OR Test-Tube Fertilization OR Fertilization, Test-Tube OR Fertilizations, Test-Tube OR Test Tube Fertilization OR Test-Tube Fertilizations OR Fertilizations in Vitro OR Test-Tube Babies OR Babies, Test-Tube OR Baby, Test-Tube OR Test Tube Babies OR Test-Tube Baby)

7：TI=(Insemination, Artificial OR Eutelegenesis OR Eutelegeneses OR Artificial Insemination OR Artificial Inseminations OR Inseminations, Artificial) OR AB=(Insemination, Artificial OR Eutelegenesis OR Eutelegeneses OR Artificial Insemination OR Artificial Inseminations OR Inseminations, Artificial)

Final search strategy: (1) and (2 or 3) and (4 or 5 or 6 or 7).
